# Efficient and reproducible generation of high-expressing, stable human cell lines without need for antibiotic selection

**DOI:** 10.1186/1472-6750-8-13

**Published:** 2008-02-12

**Authors:** Gudrun Schiedner, Sabine Hertel, Corinna Bialek, Helmut Kewes, Gero Waschütza, Christoph Volpers

**Affiliations:** 1 CEVEC Pharmaceuticals GmbH, Gottfried-Hagen-Str. 62, 51105 Cologne, Germany

## Abstract

**Background:**

Human cell lines are the most innovative choice of host cell for production of biopharmaceuticals since they allow for authentic posttranslational modification of therapeutic proteins. We present a new method for generating high and stable protein expressing cell lines based on human amniocytes without the requirement of antibiotic selection.

**Results:**

Primary amniocytes from routine amniocentesis samples can be efficiently transformed with adenoviral functions resulting in stable human cell lines. Cotransfection of the primary human amniocytes with a plasmid expressing adenoviral E1 functions plus a second plasmid containing a gene of interest resulted in permanent cell lines expressing up to 30 pg/cell/day of a fully glycosylated and sialylated protein. Expression of the gene of interest is very stable for more than 90 passages and, importantly, was achieved in the absence of any antibiotic selection.

**Conclusion:**

We describe an improved method for developing high protein expressing stable human cell lines. These cell lines are of non-tumor origin, they are immortalized by a function not oncogenic in human and they are from an ethically accepted and easily accessible cell source. Since the cell can be easily adapted to growth in serum-free and chemically defined medium they fulfill the requirements of biopharmaceutical production processes.

## Background

For many therapeutic proteins post-translational modifications, proteolytic processing and oligomerization of multiple chains are important for protein functionality, stability and efficient secretion. Even though some modifications can occur in yeast and bacterial expression systems, mammalian and preferably human cells are the host of choice for proteins that require authentic glycosylation or other post-translational modifications. For production of biopharmaceuticals, there is a permanent demand for improved methods for cell line development featuring shorter time lines, higher productivity, improved consistency and genetic stability.

Rapid production of small quantities of protein can be achieved by transient transfection of the appropriate mammalian cell line. In contrast, large-scale protein production depends on a stable cell line with the protein expressing genetic construct integrated in the host genome. The development of a permanent production cell line and the manufacturing process for a recombinant protein usually follows a well-established scheme. For screening purposes and for maintenance of protein expression potent selection markers need to be used and producer cells have to be constantly cultivated in medium containing the respective selective agent. The two expression cassettes – one expressing the protein of interest, the second containing the selection marker – can either be located on different plasmids, or can be incorporated in one plasmid, preferably expressed from the same promoter by taking advantage of an internal ribosomal entry site (IRES) [1]. By transfection and subsequent constant selection in the appropriate selection medium producer cell lines are selected which express high levels of the protein of interest. Classical selection markers like glutamine synthetase (GS) [2,3], dihydrofolate reductase (DHFR) [4,5], hypoxanthine guanine phosphoribosyl transferase (HPRT) [6,7] or herpes simplex virus TK [8,9] genes can only be used in cells deficient for the respective gene. Alternatively, genes that confer resistance to cytotoxic drugs can be used like kanamycin, neomycin, geneticin and blasticidin [10].

Usually, the development and selection of an optimized permanent producer cell line is a very time-consuming procedure which can last for months and includes selection for cell clones with highest expression levels, limited dilution to obtain genetically identical cell clones and testing for stability of expression during multiple passages. Expression levels of the protein of interest depend on numerous factors including the promoter, cellular levels of relevant transcription factors, presence of factors transactivating the promoter, the number of gene copies within the cell and the chromatin structure at the integration site [11].

The site of integration has a major effect on the transcription of the gene of interest. Therefore, integrations into transcriptional active chromatin sites are preferred. However, very frequently the gene of interest is rapidly inactivated and thus silenced [12,13]. Several strategies to overcome this position effect have been developed and include the use of regulatory elements like matrix attachment regions and insulators flanking the gene of interest [14]. In addition, specific targeting of the gene of interest into transcriptionally active sites of the genome using yeast or phage recombinases seems to be a possible option [15,16].

The production of authentic human proteins is best addressed by the use of human cell lines, because they are not expected to add potentially immunogenic glycan structures to the protein of interest. However, there are only a few human cell lines described and restricted access or deficient documentation limit their use in biopharmaceutical production. Among the cell lines used are HEK293 (E1-transformed human embryonal kidney/neuronal cells) [17], HKB11 (HEK293 cells fused with a Burkitt's lymphoma cell) [18], PerC6 (E1-transformed human embryonal retina cells) [19], and E1-transformed human amniocyte cells [20,21].

We describe here a novel method for rapid generation of human production cell lines capable of secreting high levels of proteins without any need for antibiotic selection. Our approach was to co-transfect primary human amniocytes with two plasmids, one expressing adenoviral E1-gene products and a second expressing a therapeutic protein. Transformed cell clones were obtained which show high and stable expression of the glycosylated therapeutic protein. Development of these cell lines did neither require genetic knock-out of internal marker genes, nor cotransfection of a selection marker, nor selection of clones in medium containing antibiotics.

## Results

### Primary amniocytes can be efficiently transformed by adenoviral E1-functions

In order to test if primary amniocytes can be used to establish permanent, therapeutic protein expressing cell lines, amniocytes were cotransfected with the E1-expressing plasmid pGS119 containing E1A-, E1B- and pIX-functions plus the plasmid pGS116 expressing the human alpha-1 antitrypsin (hAAT) glycoprotein.

The primary amniocytes used here originated from a single amniocentesis sample. About 20–30 days after transfection of approximately 1 × 10^6 ^cells, numerous transformed cell colonies appeared on all dishes, and cells on the dishes were expanded as 10 pools (Z171-1A, B – Z171-5A, B). Non-transformed cells stop growth already at early passages and in approximately passage 10–15 mainly transformed cells can be detected on the dishes. Since transfection efficiency of primary cells is less than 1% (data not shown) the efficiency of transformation is very high, however, because cells were passaged twice the number of independently transformed clones could not be determined. Such high frequencies were obtained without difference in numerous transfections using different cells from several amniocentesis (data not shown).

**Figure 1:**
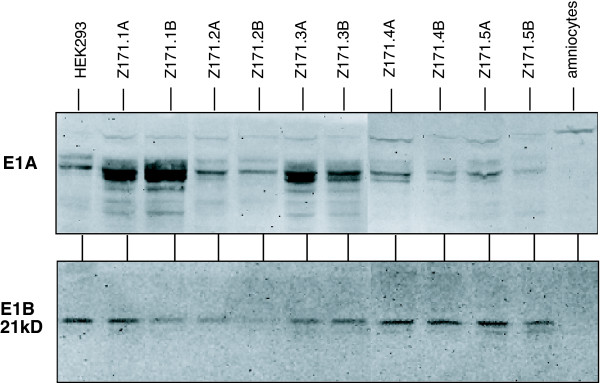
**Expression of E1 proteins**. Western Blot analyses for E1A and 21-kDa E1B proteins expressed in 10 E1-transformed amniocyte cell pools. Cells were lysed and proteins were size fractionated on a SDS-containing polyacrylamide gel. Proteins were transferred to nitrocellulose and probed with anti-E1A or anti-E1B 21-kDa antibody. Protein lysates from HEK293 or primary amniocytes were used as control.

Expression of the E1 proteins was analyzed by Western Blot using protein extracts of different cell pools. Like HEK293 cells used as positive control, all cell pools express both the E1A- and 21kD-E1B proteins, although in different amounts and ratios (Fig. 1).

Primary amniocytes show variable morphologies. In contrast, transformed cells do appear smaller and show more uniform morphologies. In very early passages the morphologies in different cell pools are quite similar but change during further cultivation. Most transformed cells go through mild crisis and decelerate growth but recover during only few passages.

### Transformed amniocytes express and secrete hAAT

**Figure 2:**
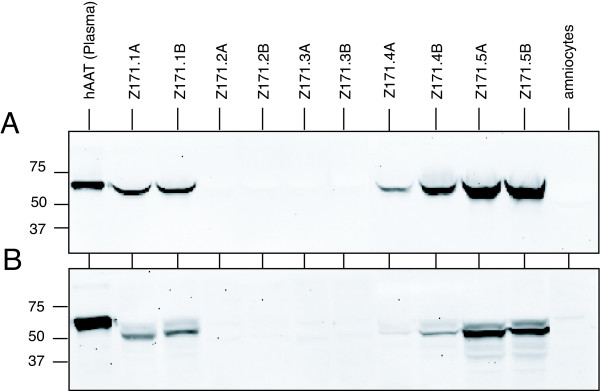
**Expression of hAAT protein**. Western Blot analyses for hAAT protein expressed in 10 E1-transformed amniocyte cell pools. (A) For detection of secreted hAAT, proteins in the cell culture medium were fractionated on a SDS-containing polycrylamide gel. (B) For detection of intracellular hAAT, cells were lysed and proteins were separated on a SDS-containing polyacrylamide gel. Intracellular and secreted hAAT was visualized using a monoclonal anti-hAAT antibody. For control proteins from untransformed amniocytes and hAAT purified from human plasma were used.

As described above, the primary amniocytes were cotransfected with a hAAT-expressing plasmid. hAAT is a major human serum protein which is predominantly produced and efficiently secreted from hepatocytes. Thus we tested for secreted (Fig. 2a) and intracellular (Fig. 2b) hAAT in different cell pools. Using Western Blot analyses we were able to detect hAAT expression in 6 out of 10 different cell pools. The amount of protein loaded per lane for the intracellular and secreted protein corresponds to 8 × 10^4 ^cells and 4 × 10^3 ^cells, respectively. These results suggest that hAAT is very efficiently secreted from the cells since only small amounts of the protein were intracellularly located.

### Amniocyte cell lines show high and long-lasting hAAT expression

The above results show that both plasmids integrated upon cotransfection and cells express proteins from both plasmids. The lack of selective pressure during cell passaging most likely does not influence expression of E1-functions since their continuous presence is expected to be crucial to maintain the transformed phenotype of the cells. Therefore, we tested for the stability of expression of hAAT during multiple passages.

**Figure 3:**
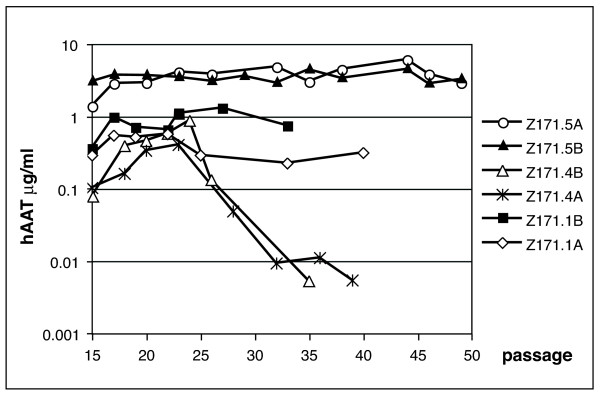
**Quantitation of hAAT expression in 6 amniocyte cell pools**. Cells in different passages were plated into 6-well plates, the supernatants were collected after 72 hours and the amounts of secreted hAAT were analysed by ELISA.

Six Z171 cell pools showed expression of hAAT in early passages and were thus further cultivated up to 35–50 passages. In different passages 7 × 10^5 ^cells were plated, the supernatants were collected and the amounts of secreted hAAT were quantitated. In figure 3 the amount of hAAT in 6 different cell pools is depicted and shows that 4 out of 6 cell pools show high and long-lasting hAAT expression up to 6 μg/ml. Only in two cell pools the hAAT expression drops drastically to almost not detectable expression levels after 30 passages. Two cell pools (Z171-5A and Z171-5B) stably express up to 8 pg hAAT per cell and day for more than 50 passages without any antibiotic selection.

**Figure 4:**
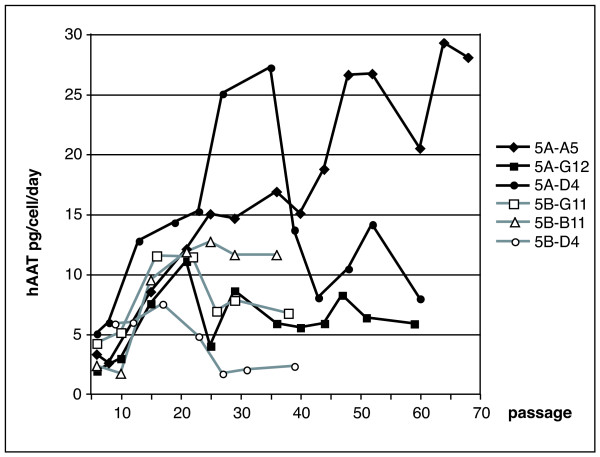
**Quantitation of hAAT expression in 6 clonal amniocyte cell lines**. Single cell cloning was performed from amniocyte cell pools Z171-5A and Z171-5B in passages 26 and 27, respectively. Clonal cells in different passages were plated into 6-well plates, the supernatants were collected after 48 hours and the amounts of secreted hAAT were analysed by ELISA.

Since each cell pool is derived from numerous different transformation and integration events and thus contains many genetically different cell lines, we performed single cell cloning on cell pools Z171-5A and Z171-5B by limited dilution in 96-well plates. Thirteen single cell clones originating from Z171-5A and 25 single cell clones originating from Z171-5B were expanded and tested for hAAT expression (data not shown). Figure 4 shows long term expression of hAAT of 3 clonal cell lines each originating from pool Z171-5A and Z171-5B, respectively. Over 60 passages all 6 clonal cell lines express hAAT; in 2 clonal lines maximum hAAT levels reach 27 and 30 pg/cell/day.

### hAAT expressed in amniocyte cell lines is glycosylated and sialylated

**Figure 5:**
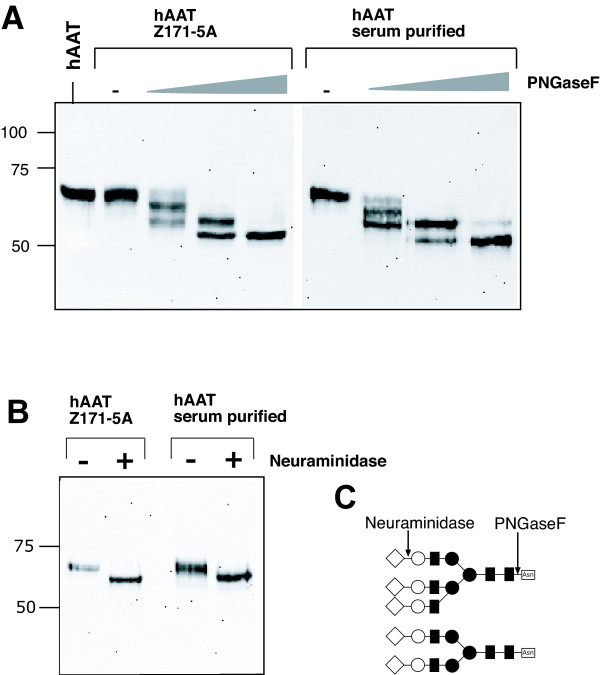
**Glycoanalyses of hAAT**. Proteins from cell culture medium of amniocyte cell pool Z171-5A were treated with increasing amounts of (A) PNGaseF or with (B) Neuraminidase and fractionated on SDS-containing polyacrylamide gels. Upon transfer on nitrocellulose Western Blot analyses were performed using a monoclonal anti-hAAT antibody. For controls hAAT purified from human plasma was used. (C) A- and B-type oligosaccharide chains of hAAT and cut positions of PNGaseF and Neuraminidase.

Human alpha-1 antitrypsin is a 396- amino acid serum glyoprotein and contains three carbohydrate side chains N-linked to asparagine residues. Analyses of the carbohydrate composition revealed two main A- and B-types oligosaccharide chains (see Fig 5c) in 2:1 ratio [22,23]. Glycosylation of hAAT does not seem to be important for the formation of a biologically active conformation with elastase and thus for activity of the protein [24]. However, glycosylation seems to play a crucial role in stability of hAAT in the serum [25,26] and in secretion from hepatocytes [27].

In order to test if amniocyte cell lines produce glycosylated hAAT we treated the cell culture supernatants of Z171-5A (Fig. 5) and Z171-5B (data not shown) with PNGaseF, followed by hAAT-specific Western Blotting. Since PNGaseF cleaves between the innermost GlcNAc and asparagine residue of oligosaccharides from N-linked glycoproteins (see Fig. 5c) we expected a shift in molecular weight if PNGaseF can hydrolyze N-glycan chains from hAAT. For control we used hAAT purified from human serum. Digestion with increasing amounts of PNGaseF results in a clear shift in molecular weight (Fig 5a). Moreover, appearance of three distinct bands clearly suggests the presence of three glycan chains in the recombinant product. There is no difference in size and number of different protein bands when compared to hAAT purified from human serum.

All galactoside residues in plasma derived hAAT are known to be linked with sialic acid. To test for this feature in hAAT expressed in amniocyte cell lines, protein secreted into the cell culture medium of Z171-5A was digested with Neuraminidase followed by hAAT-specific Western Blotting (Fig. 5b). Comparable to plasma derived hAAT, the hAAT produced in Z171-5A showed a shift in size when treated with Neuraminidase.

These results indicate that hAAT produced in human amniocyte cell lines are fully glycosylated and sialylated.

## Discussion

Increased and detailed knowledge about the precise carbohydrate structures has revealed the importance of post-translational modifications for functionality of therapeutic proteins. However, these findings also exposed that the list of modifications is frighteningly long and many of them might affect the immunogenicity, stability, pharmacokinetics and thus efficacy of the protein. The most important capability that distinguishes mammalian cells from other expression systems is N- and O-linked glycosylation and it is assumed that around 2% of the human genome encodes proteins that contribute to glycosylation [28]. Although almost any mammalian cell line possesses the machinery to produce and secrete proteins, only a limited number meet the fermentation requirements and thus can be used for industrial manufacturing: Chinese hamster ovary (CHO), baby hamster kidney (BHK) and mouse myeloma cells NS0 and Sp2/0.

Glycan structures differ significantly among different cell types and species. Only human cells lines promise to produce proteins in a way that species-specific and thus immunogenic differences in glycosylation are absent. For safety and regulatory reasons human cells used for production should not be of tumor origin. In addition, only a limited number of human cell lines are available to date. The development of permanent cell lines using primary human cells and recombinant DNA techniques has been hampered by the fact that human cells are highly resistant to transformation by viral functions. In fact, from human tissue sources only human embryonic kidney (HEK) cells [17], human embryonic lung (HEL) cells [29], human embryonic retinoblasts (HER cells) [30,19] and primary human amniocytes [20] have been successfully transformed with adenoviral functions. For practical and ethical reasons it is very difficult to obtain primary cells from fetal origin. Primary human amniocytes are the only cell type that is readily available without ethical concerns: they can be collected by routine amniocentesis. Human amniocytes can be cultivated as adherent cultures for several passages under standard conditions and consist of three main cell types, fibroblast-like, epithelial-like and amniotic fluid (AF) cells [31]. Recently it has been shown, that about 1% of cells found in amniotic fluid are amniotic-fluid derived stem (AFS) cells [32]. Considering the low transfection efficiency (< 1% in the present analyses) of human amniocytes, the transformation efficiency using an E1-expressing plasmid is surprisingly high. In earlier studies we have calculated to obtain at least one or two transformants in 1 × 10^5 ^cells transfected [20]. Even though in the present analyses we have transfected two plasmids, one containing the E1 and pIX genes and a second expressing hAAT with a plasmid ratio of 1:1, the transformation efficiency was comparable to transfection with the E1-plasmid alone (data not shown).

Cotransfection of two plasmids expressing the transforming E1-functions and hAAT, respectively, resulted in stably transformed cell pools, with 6 out of 10 pools expressing hAAT. Moreover, 4 out of 6 cell pools show long-lasting expression for more than 35 passages, with expression levels up to 6 μg/ml or 8 pg/cell/day. Cell cloning by limited dilution from two cell pools resulted in genetically identical cell lines which show stable expression of hAAT for more than 65 passages (the time course of the present study) with expression levels up to 30 pg/cell/day. Since cloning of cells was started in passage 26, this indicates stable expression of hAAT for more than 90 passages. Again it has to be emphasized that this stable protein expression was achieved without any antibiotic selection.

The majority of the mammalian genome is transcriptionally silent. Since integration of transfected plasmids occurs randomly, the position effects generally manifest partial or complete loss of expression. In order to overcome the position effect numerous regulatory elements introduced into the plasmid expressing the gene of interest have been tested including insulators, MARs, strong promoters and enhancers. Even when using such elements a time consuming testing of numerous cell clones over multiple passages cannot be avoided. Other crucial parameters like the choice of promoters for expressing the gene of interest and the marker gene, the ratio of gene of interest to marker gene expressing plasmid, concentration of selection marker in the medium or using linear versus circular plasmids considerably influence cell line development and expression levels.

In common strategies to select for highly expressing cell lines, the marker gene is linked to the gene of interest and employing a selection strategy can circumvent the problem of silencing. However, even under selective pressure only a very minor number of cell clones yield in high and stable expression of the gene of interest. In addition, the selective pressure has to be maintained throughout cell line development. Currently, aminoglycoside antibiotics such as hygyromycin B and Geneticin are frequently used. These substances interfere with protein translation and exhibit highly toxic effects in mammalian cells not containing the corresponding bacterial genes. However, these antibiotics are also described to have undesired side effects like increasing frequency of sister chromatid exchange [33] and altering expression of glucose-regulated genes [34].

A different very popular strategy for high-level protein production is based on the use of *dhfr*-deficient CHO cells in combination with expression vectors carrying a functional *dhfr *gene and the gene of interest. Cultivation of these transfected cells in methotrexate (MTX) containing medium results in amplification of the *dhfr *gene and gene of interest sequences and thus high-level expression of the gene of interest. The disadvantage of this production system however is, that cells grown in the presence of MTX often show substantial heterogenicity in chromosomal location and copy number of amplified sequences, they exhibit rearrangement and highly variable amplification of transfected sequences, and they contain chromosomes with highly extended regions, chromosomes joined at amplified regions or even circular chromosomes consisting entirely of exogenous DNA. Thus the use of the *dhfr*-amplification system very often results in a high degree of genetic instability in the production cell line [35].

As described earlier, the permanent expression of E1-functions is crucial for maintaining the transformed character of stable cell lines [36]. Thus, the E1-functions are replacing the selection marker and prevent the repressive effect of the surrounding heterochromatin. Earlier analyses have shown, that transfection of two plasmids into CHO cells results in co-integration at a common site in all clones examined [37]. Since in the present analyses 4 out of 6 cell pools show long-lasting expression of hAAT, we assume that the hAAT-expressing plasmid has co-integrated in a transcriptionally active site. Single cell cloning of the cell pools resulted in stable cell lines exhibiting very high protein expression of up to 30 pg/cell/day. The E1-functions have been shown to increase expression from several promoters including the CMV promoter [38-40] used in the present analyses, which might contribute to the high expression levels.

Primary human amniocytes are efficiently transformed by adenoviral E1-functions. Based on this observation, we exhibit an improved method for developing high protein expressing stable human cell lines. These cell lines can be easily adapted to serum-free suspension culture by gradually exchanging the medium to a serum-free, chemically defined medium for suspension cells (data not shown). Moreover, hAAT expressed as reference protein in human amniocyte cell lines is fully glycosylated and sialylated. In future experiments we plan to perform a more detailed analyses of the glycan structure of hAAT expressed in human amniocyte cell lines in comparison to the protein expressed in CHO cells.

Industrial protein expression demand short time lines for cell line development, use of chemically derived serum free medium, growth in suspension and the possibility to scale up production process. Only during the very early passages the primary amniocytes depend on fetal serum but are soon transferred to chemically derived serum-free medium. For technical reasons transformation and isolation of permanent cell clones occurs in adherent culture, and thus an additional step for adaptation to growth in suspension has to be admitted. We have started additional experiments in order to simplify and speed up this adaptation step and thus shorten the time frame for cell line development.

The potential of the present novel method of cell line development however is not restricted to production of biopharmaceuticals. For example, cotransfection of primary amniocytes with the E1-expressing plasmid and a second plasmid expressing SV40 T-antigen or Epstein-Barr virus EBNA-1 protein would result in cell lines that would be optimized for transient protein expression. Moreover, overexpression of glycosylation enzymes like the α2–6 sialyltransferase would result in cells optimized for production of glycosylated proteins with high sialic acid content. Additional examples would be the expression of certain viral proteins in cells lines for improved production of viruses for vaccination or gene therapy. The current method would also allow the insertion of certain DNA-sequences like FLP recombinase targets (FRT) sites by simply introducing FRT sites in the E1-expressing plasmid. By cotransfecting such new cells with a plasmid expressing the gene of interest flanked by FRT sites and a FLP expressing plasmid, a recombinase mediated cassette exchange would occur. The insertion of the gene of interest would then occur at a predicted site and thus would drastically simplify cell line development.

## Conclusion

We have shown that cotransfection of primary amniocytes with two plasmids, one expressing transforming adenoviral E1-functions and the second plasmid expressing hAAT as a gene of interest, resulted in high hAAT-expressing cell lines. In this new human cell lines protein expression of up to 30 pg/cell/day was stable for more than 90 passages and was accomplished without antibiotic selection. Moreover, the hAAT produced in the new amniocyte cell lines was fully glycosylated and sialylated. This new method shows an efficient way of developing stable and human cell lines from a non-tumorgenic, ethically accepted and easily available cell source.

## Methods

### Plasmids

Plasmid pGS116 contains the human cytomegalovirus (hCMV) promoter, followed by a Simian virus 40 (SV40) intron, the human alpha-1 antitrypsin (hAAT) cDNA and the SV40 polyadenylation site. Plasmid pGS119 was used to transform primary human amniocytes and contains the E1 and pIX region of adenovirus type 5 (Ad5) from nt. 505 to 4079. E1A is under the control of the murine phosphoglycerate kinase (pgk) promoter, while E1B and pIX expression is controlled from their natural promoters. The E1B downstream intron, splice acceptor and polyA signal were replaced by corresponding motifs from SV40.

### Primary cells and cell lines

Primary amniocytes were derived from routine clinical amniocentesis usually performed during week 16–20 of gestation for prenatal diagnosis. Amniocytes were obtained including patients informed consent as redundant material not required for clinical diagnosis. Thus, the amniotic fluid was used in accordance with all legal and ethical requirements. Primary cells were cultivated in Ham's F10 medium supplemented with L-glutamine (Invitrogen), 10% fetal calf serum (FCS), 2% Ultroser G (BioSpepra) antibiotic/antimycotic (Invitrogen) in 5% CO_2 _at 37°C. After several passages cells were stepwise adapted to Opti-Pro medium (Invitrogen), supplemented with Ultroser. HEK-293 cells were cultivated in alpha modified Eagle's medium (αMEM, Invitrogen) supplemented with 10% FCS and antibiotics in 5% CO_2 _at 37°C.

### Transfection and expansion of transformed amniotic cells

Five 6-cm dishes with subconfluent cultures of amniocytes adapted to Opti-Pro medium (see above) were transfected with 1 μg per dish of each pGS116 and pGS119 using Effectene transfection kit (Qiagen). Both plasmids were digested with ScaI prior to transfection. The next day, transfected cells were transferred to 15-cm dishes and Ultroser was reduced to 1%. About 20 to 30 days post transfection cells from each dish were transferred to two 15-cm dishes; individual cell clones arising from single transformed cells appear on the transfected dishes several days later. These clones were expanded as clone pools by passaging the cells on individual dishes and further cultivation for several passages until untransformed amniocytes stopped growing and were overgrown by transformed cells. Genetically identical clones of two pools of Z171 in passage 26 (for Z171-5A) and 27 (for Z171-5B) were isolated by limited dilution in 96-well plates and further expanded. Passage numbering of cloned cells started again with passage one in 96-well plates.

### Expression of E1 proteins

For detection of E1 protein expression Western Blots were performed using monoclonal antibodies. 7 × 10^5 ^cells/well from Z171 cell pools were plated on 6-well plates and harvested 72 h later by detaching the cells in Tris-Saline/4 mM EDTA, pelleting and lysing cells in loading buffer containing SDS. For control either untransfected primary amniocytes or untransfected HEK293 cells were used. The proteins were separated in a 10% SDS-polyacrylamid gel, transferred to nitrocellulose and incubated with either an anti-E1A or an anti-E1B-21kD antibody (Oncogene Research), and anti-mouse (E1A, Jackson ImmunoResearch Laboratories) or anti-rat (E1B-21kD, Oncogene Research) as secondary antibody and visualized by chemoluminescence.

### Expression of hAAT (Western Blot)

The intracellular and secreted hAAT expression in different cell lines were analysed in a Western Blot using a monoclonal antibody. 7 × 10^5 ^cells/well from each Z171 cell pool were plated on 6-well plates. Seventy-two hours later the supernatants were collected, cells were counted and lysed as described above. For detection of intracellular expression of hAAT proteins, cell lysates were separated on a 10% SDS-Gel. For detection of secreted hAAT, 10 μl of the cell culture media were applied on a 10% SDS-gel. Intracellular and secreted hAAT was visualized by chemoluminescence using a monoclonal anti-hAAT antibody (ICN Biomedicals), and an anti-goat antibody (Pierce). As negative controls, proteins from untransfected primary amniocytes were used. As positive control, hAAT purified from human plasma (ICN Biomedicals) was used.

### Quantitation of hAAT expression (ELISA)

The amount of hAAT secreted into the cell culture medium was quantitated using the enzyme-linked immunoabsorbent assay (ELISA). From different passages of each Z171 cell pool 7 × 10^5 ^cells/well were plated on 6-wells plates. Forty-eight or 72 hours later the supernatants of the different cell lines were collected. The amount of hAAT secreted into the medium was quantitated by ELISA using polyclonal hAAT-antibodies (HRP-coupled and uncoupled, ICN Biomedicals). As standard, different amounts of hAAT purified from human plasma were used. The hAAT levels were either calculated in μg expressed per ml medium or as pg/cell expressed in 24 h per cell using the numbers of cells initially plated.

### Glycoanalyses of hAAT

The glycosylation of hAAT expressed in Z171-5A and Z171-5B was analysed by cleaving N-linked oligosaccharides from hAAT using the peptide-N-glycosidase F (PNGaseF). To 22.5 μl cell culture medium, 2.5 μl of denaturing buffer (5% SDS, 10% β-Mercaptoethanol) was added. After denaturing for 10 min at 96°C, 3.5 μl G7-reaction buffer (New England Biolabs, 10× containing 500 mM Na-phosphate buffer), 3.5 μl 10% NP-40 and different amounts of PNGaseF (500, 50, 5 and 0 U/μl, New England Biolabs) were added and incubated for 1 h at 37°C. For control either hAAT purified from human plasma or 22.5 μl of cell culture medium were denatured and incubated either with PNGaseF or H_2_O as described above.

For detection of sialic acid linked to galactose residues, hAAT secreted into the cell culture medium was incubated with Neuraminidase. This enzyme catalyzes the hydrolysis of α2–3, α2–6 and α2–8 linked N-acetyl-neuraminic acid residues from glycoproteins. To 22.5 μl cell culture or medium plasma derived hAAT, 2.5 μl G1-reaction buffer (New England Biolabs, 10× containing 500 mM sodium citrate) and 3 μl Neuraminidase (50 U/μl, New England Biolabs) or 3 μl H_2_O was added and incubated for 1 h at 37°C.

Controls and digested proteins were separated in an SDS-polyacrylamide gel and Western Blotting using anti-hAAT antibodies was performed as described above.

## Authors' contributions

GS designed and coordinated the study and drafted the manuscript. SH, CB and HK performed the experimental work. CV participated in the draft of the manuscript. All authors read and approved the final version of the manuscript.
